# Development of a Novel Immune Gene Array to Assess Mosquito Gene Expression During Arbovirus Infection

**DOI:** 10.3390/v18080818

**Published:** 2026-07-25

**Authors:** Juliette Lewis, Dahlia Kopycienski, Dana Mitzel, Rebekah C. Kading

**Affiliations:** 1 Department of Microbiology, Immunology and Pathology, College of Veterinary Medicine and Biomedical Sciences, Center for Vector-Borne Infectious Diseases, Colorado State University, Fort Collins, CO 80523, USA; julietterdlewis@gmail.com (J.L.); dahliakpy@gmail.com (D.K.); 2 Foreign Arthropod-Borne Animal Diseases Research Unit, Agricultural Research Service, United States Department of Agriculture, 1515 College Ave., Manhattan, KS 66502, USA; dana.mitzel@usda.gov

**Keywords:** bunyavirus, vector competence, transcriptomics, RNA interference, immunity, emerging viruses, Rift Valley fever virus

## Abstract

Assessing mosquito vector competence for emerging arboviruses, including the underlying determinants of infection, is a foundational line of investigation towards understanding arbovirus transmission dynamics and vector–virus interactions. We developed a novel quantitative immune gene array that measures the relative expression of 22 immune genes with functional representation across five major immune pathways using quantitative reverse-transcriptase polymerase chain reaction. Homologous primers were developed for *Culex tarsalis*, *Cx. quinquefasciatus*, and *Ae. aegypti* mosquitoes. As an initial pilot test for this array, *Cx. tarsalis* and *Ae. aegypti* mosquitoes were orally challenged with Rift Valley fever virus (RVFV) MP12 strain. Midguts, legs/wings, and salivary glands were harvested at 3 and 7 days post-challenge. Differential gene expression was measured against *Rpl32* as a housekeeping gene. This assay was sensitive enough to generate results for single mosquito midgut and salivary gland tissues, providing an initial snapshot of the types of gene expression patterns that can be observed for two mosquito species infected with MP12. This new tool will be broadly applicable for assessing mosquito immune profiles for arbovirus infection across major immune genes in five key pathways, as well as generating comparable results across mosquito and arbovirus systems.

## 1. Introduction

A number of factors intrinsic to the mosquito govern vector competence [1]. For a virus to be transmitted by a mosquito, it must overcome four physical barriers as it transits through the mosquito. First, it must be able to infect the midgut upon being imbibed in a bloodmeal and subsequently escape the midgut into the hemolymph. Once in the hemolymph, the virus travels to the salivary glands, where it again must infect and then escape this tissue barrier to be expectorated in saliva during a subsequent bloodmeal from a new host. For this reason, the midgut and salivary glands are considered the two major infection/escape barriers in a mosquito. The strength of these barriers is a determinant of vector competence.

The immune response is recognized for its key role in moderating viral infection in mammals, yet it remains understudied as a determinant of infection/escape barrier strength and thus vector competence in mosquitoes [1]. The number of studies using transcriptomic approaches to study gene expression in mosquitoes is increasing [2,3,4,5,6], particularly with recent advances in the technology and the availability of annotated genomes from different mosquito species. In particular, transcriptomic analysis has provided valuable insights into the vector competence of *Ae. aegypti* mosquitoes [2,3]. However, the use of many different models (cells from different species/tissues, different mosquito species, sample types, pathogens, time points, etc.) and techniques (RNAseq, RNAi knockdown, qPCR)makes it challenging to draw firm conclusions and extrapolate to other virus systems. Some studies focus on individual immune genes or pathways or a particular vector–virus association. Moreover, comprehensive and informative techniques such as RNAseq can be cost-prohibitive and computationally challenging for some laboratories to use. The availability of an assay that is broadly applicable across mosquito species and that provides data for several immune pathways simultaneously would be a valuable tool for assessing mosquito immune responses more broadly in association with vector competence for emerging arboviruses.

Rift Valley fever phlebovirus (RVFV; order *Bunyavirales*, family *Phenuiviridae*) is a mosquito-transmitted, high-consequence pathogen affecting both humans and livestock [7,8,9,10,11]. Human illness from infection with RVFV is usually febrile, but can progress to hepatitis, encephalitis, retinitis, vision loss, jaundice, severe anemia, neurologic manifestations, renal failure, and hemorrhagic fever [7,8,9]. RVF outbreaks are characterized by “abortion storms” among livestock, during which mortality rates can reach 100% in young and neonatal animals, and 10–20% in adult ruminant livestock [10,11]. Historically, RVFV has been restricted to the African continent; however, significant outbreaks have spread to the Arabian Peninsula and Madagascar [9,12,13], presenting a changing context of viral ecology. As globalized travel increases and human populations shift in response to natural disasters and political unrest, this threat will grow. Previous work has shown that many mosquito species in the United States can support replication and transmission of RVFV [14,15,16,17], and introduction to new geographic areas could occur through infected travelers [18,19,20]. Thus, it is of paramount importance to understand the factors that determine the vector competence of North American mosquito species for RVFV to be prepared for an introduction event.

In this work, we developed a qPCR array for assessing mosquito immune gene responses to virus infection and validated this array for two important North American mosquito species that can transmit RVFV in the lab: *Aedes (Stegomyia) aegypti* [L.] and *Culex (Culex) tarsalis* Coquillett. *Culex tarsalis* is generally appreciated as a more efficient vector of RVFV than *Ae. aegypti* under experimental conditions in our laboratory [21]. The array targets genes in five key innate immune pathways in the mosquito: JAK-STAT, IMD, Toll, JNK, and RNAi. By strategically targeting the ligands, receptors, enzymatic components, regulators, and response genes associated with each pathway, we aimed to decipher which pathway(s) are playing a role in controlling viral dissemination in *Ae. aegypti* and *Cx. tarsalis* mosquitoes. Here, to test the performance and utility of this qPCR array in vector competence experiments, we conducted a pilot experiment with *Ae. aegypti* and *Cx. tarsalis* mosquitoes challenged with the RVFV MP12 strain. We sought to gather preliminary data on the innate immune response in the midgut and salivary glands observed for *Ae. aegypti* with no detectable dissemination with RVFV MP12 and *Cx. tarsalis* with detectable dissemination of RVFV MP12 in the lab. These investigations serve as an initial assessment of assay feasibility and a means to generate hypotheses regarding species and tissue-specific immune expression patterns following virus challenge.

## 2. Materials and Methods

### 2.1. Primer Design and Validation

**Figure 1:**
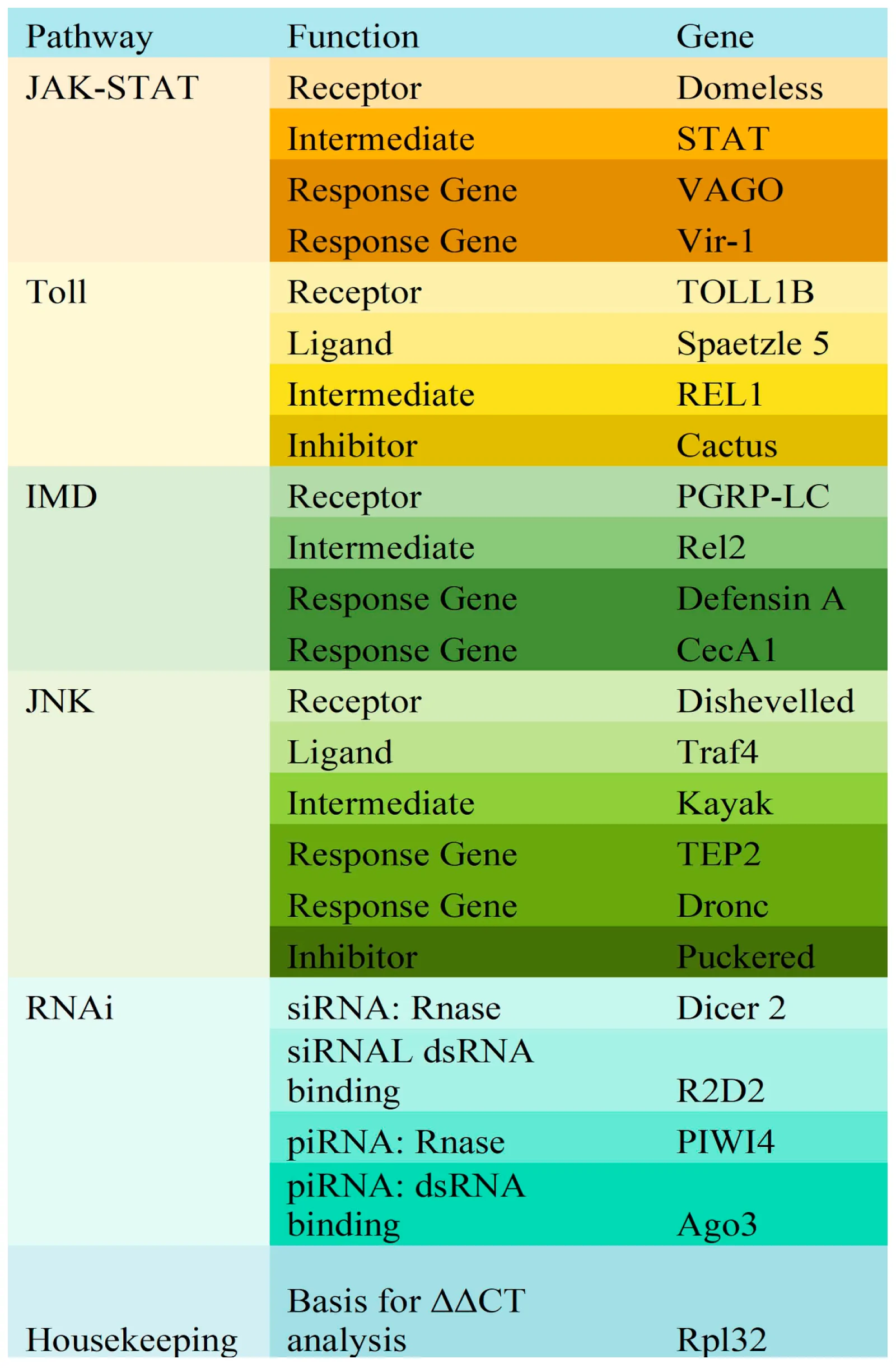
Genes and corresponding function in each immune pathway selected for qPCR array.

Five major mosquito immune pathways were examined for potential qPCR array targets based on the role of the target and, if available, published evidence for differential expression of that target in a relevant model system. For *Cx. tarsalis*, due to the species’ unannotated genome, gene sequences of interest were identified by blasting known *Cx. quinquefasciatus* gene sequences across the entire *Cx. tarsalis* genome. Candidate sequences from *Cx. tarsalis* were then aligned with the known *Cx. quinquefasciatus* sequences. This iterative process was done to validate sequence identity as well as to design *Cx. tarsalis* primers in conserved genetic regions across both species. Primers were then designed to target a 100–150 bp amplicon in the mRNA for the gene of interest in each species, including *Cx. quinquefasciatus* (Supplementary Tables S1 and S2; Zenodo repository) and included multiple targets and functional roles in each of the major immune pathways (Figure 1). For *Ae. aegypti*, since a fully annotated genome was available, primers were designed directly using sequences available on VectorBase (Supplementary Table S3). Primers were blasted in silico to check for potential off-target binding. Primers were validated using RNA extracted from individual midguts and salivary glands of the relevant species and underwent melting curve analysis to again check for off-target effects.

### 2.2. Mosquito Rearing

*Culex tarsalis* (strain KNWR) and *Ae. aegypti* (Poza Rica, Mexico) mosquitoes were reared and held in a controlled environment at 28 °C, 70% humidity, and a 16:8 (*Cx.*) or 12:12 (*Ae.*) light–dark cycle. During colony pupation, 500 mosquitoes were collected as pupae and housed in 64 oz screen-top paperboard cartons for each experimental group (uninfected, and day 3 and 7 post-challenge with MP12). Approximately 16–18 h prior to blood meal, the sugar source (raisins) were removed from cartons to starve mosquitoes and encourage blood feeding. The water source was removed 2–4 h prior to blood feeding.

### 2.3. Cells and Viruses

Vero cells (ATCC CCL-81, Manassas, VA, USA) were cultured in complete Dulbecco’s modified Eagle’s medium (DMEM; Corning, New York, NY, USA), supplemented with 5% or 10% fetal bovine serum (R&D Systems, Minneapolis, MN, USA; Atlas Biologicals, Fort Collins, CO, USA) and 1% antibiotic–antimycotic solution (ThermoFisher Scientific, Waltham, MA, USA) at 37 °C with 5% CO_2_ atmosphere in a cell culture incubator. 

Freshly grown Rift Valley fever phlebovirus live attenuated vaccine strain MP12 (Genbank: DQ375404, DQ380208, and DQ380154) was spiked into artificial blood meals for oral challenge as described previously [21]. Both *Cx. tarsalis* and *Ae. aegypti* mosquitoes are susceptible to infection with and capable of transmitting MP12 at comparable rates to wild-type RVFV [21].

### 2.4. Mosquito Dissection

Virus-infected or uninfected cell supernatant was mixed in a 1:1 ratio with fresh defibrinated calf blood (Colorado Serum Company, Denver, CO, USA) and supplemented with 10 mM ATP and 0.075% sodium bicarbonate before being loaded onto a Hemotek membrane feeder (Hemotek Ltd., Blackburn, UK) and covered with hog’s gut. Membrane feeders attached to a heating unit were warmed to 37 °C and applied to cartons containing 5- to 7-day old mosquitoes starved for approximately 1 h, after which only fully engorged blood-fed females were transferred to new cartons and provided with water and raisins. Mosquito groups receiving virus-infected blood and uninfected blood were held under the same experimental conditions. Aliquots of blood were saved for back-titration.

At three and seven days post-challenge (dpc), mosquitoes were dissected in 70% molecular-grade ethanol, and the following samples were harvested: legs and wings, midguts, salivary glands, and carcasses. Immediately following dissection, samples were added to microcentrifuge tubes with TRIzol^®^ reagent (Thermofisher Scientific™, 15596026, Waltham, MA, USA) and glass beads, homogenized using a TissueLyser2 (QIAGEN^®^, Hilden, Germany) set to 30 Hz for 1 min, and stored at –80 °C. The samples were later thawed and extracted using a Direct-zol™-96 RNA kit (Zymo Research ™ R2056, Irvine, CA, USA) according to the manufacturer’s instructions.

### 2.5. RT-qPCR

The dissemination status of RVFV in mosquito samples was assessed via RT-qPCR of the legs and wings RNA [22]. The master mix for detecting MP12-positive legs and wings was made using TaqMan^®^ Fast Virus 1-Step Master Mix (Thermofisher Scientific™ Cat# 4444434, Waltham, MA, USA). RT-qPCR was performed with primers targeting the L-segment (forward primer, RVFL-2912fwdgg: TGAAAATTCCTGAGACACATGG; reverse primer, RVFL-2971revAC: ACTTCCTTGCATCATCTGATG; probe: (FAM)-CAATGTAAGGGGCCTGTGTGGACTTGTG-(BHQ1) on a QuantStudio 3 (Thermofisher Scientific™ A28137, Waltham, MA, USA) under the following cycle parameters: 50 °C for 5 min, 95 °C for 20 s, 45 cycles of 95 °C for 3 s, and 60 °C for 30 s.

Midgut and salivary gland RNA for both time points and treatments was analyzed via RT-qPCR to assess immune gene expression of the 22 validated innate immune gene primer sets (Supplementary Table S2). Four different housekeeping genes identified in the literature were analyzed to determine eligibility for use in the qPCR array as the basis for ΔΔCT analysis; actin, ribosomal protein 8 (*Rpl8*), ribosomal protein 32 (*Rpl32*), and elongation factor 1-α (EF1α). Primers were tested for each of these genes in both *Ae. aegypti* and *Cx. tarsalis* by running them on 3 biological replicates of salivary gland and midgut RNA from each species (Figure 1). All were run on the same plate at the same time to facilitate direct comparison. The ZymoScript One-Step RT-qPCR Kit (Zymo Research, Irvine, CA, USA) was used according to manufacturer instructions. RT-qPCR was performed on a QuantStudio 3 (Thermofisher Scientific™ A28137, Waltham, MA, USA), using the following cycle parameters for all genes: 55 °C for 10 min, 95 °C for 10 min, 40 cycles of 95 °C for 20 s, then 56 °C for 1 min.

### 2.6. Data Cleaning

For each time point and tissue type, data were evaluated sample-wise and gene-wise for unreliability (defined as Cq > 38). Samples were removed from analysis if >50% of genes had unreliable values. Genes were removed from analysis according to unreliability percentage separately for each species/tissue/time-point group.

### 2.7. ΔΔCT Calculation and Statistics

A subset of ten target genes were analyzed for sample size to achieve a power of ≥0.80 with significance level of 0.005 for fold changes specific to those genes. With a sample size of 6 mosquitoes per group, a power of 0.89 was achieved for detecting a fold change of 3.03 or more in Defensin D (the gene requiring the largest sample). This sample size would achieve a power of 0.89–0.99 for all ten genes tested.

Intra-species ΔΔCT was calculated as an average of data from up to six individual mosquitoes using the RQdeltaCT package (version 1.3.3) in R (version 4.5.1). The midguts and salivary glands from individual mosquitoes with (*Cx. tarsalis*) and without (*Ae. aegypti*) detectable dissemination were selected as a pilot test of the gene expression analysis. Gene expression in each of these two groups of mosquitoes was compared against mosquitoes of the same species that received an uninfected blood meal. We did not include non-blood-fed controls in these experiments. Calculated values were tested for normality by the Shapiro–Wilk test of normality. If the Shapiro–Wilk *p*-value was >0.05, then the Benjamini–Hochberg-adjusted *p*-value from the *t*-test comparison of the ΔΔCT values was used. If the Shapiro–Wilk *p*-value was <0.05, then the Benjamini–Hochberg-adjusted *p*-value obtained from the Mann–Whitney U test was used. Interspecies differences were compared using linear models. Where assumptions for a linear model could not be met (as tested by normality of residuals), then a Mann–Whitney U test was applied.

Sample quality was evaluated by looking at PCA plots for each tissue/DPI group, where each point in the graph represents a sample, and the PC score is based on a linear combination of the ΔΔCt values for that sample.

## 3. Results

### 3.1. Assay Context

*Aedes aegypti* and *Cx. tarsalis* were fed an MP12-spiked infectious (10^7^ PFU/mL) or non-infectious bloodmeal and gene expression data generated for up to six virus-challenged and six control mosquitoes for each species at days 3 and 7 post-challenge. To investigate our question of what immune genes might be contributing to the relatively strong midgut barrier in *Ae. aegypti* and a permissive barrier in *Cx. tarsalis*, mosquitoes selected for immune gene analysis included *Ae. aegypti* that did not have detectable dissemination at the two time points and *Cx. tarsalis* that did have detectable dissemination at the two time points for analysis by qPCR array. The resulting data for each mosquito species should be considered separately, and are therefore interpreted only as an initial evaluation of assay utility and the types of data that are generated, as the dissemination phenotype for the two mosquito species used for this analysis was different. Gene expression data were generated for individual tissues from each mosquito, demonstrating that this assay is sensitive enough to generate immune response data at the level of the individual tissue. To look more holistically at these pathways across mosquito species and time points, results were averaged across all individual mosquitoes.

### 3.2. Virus Dissemination Rates

Dissemination rates of MP12 were higher for *Cx. tarsalis* than for *Ae. aegypti* (Table 1), as determined by detection of RVFV RNA in legs and wings by RT-qPCR. Virus infection of the midgut and salivary gland tissues was not determined for this study due to the use of these samples entirely for the qPCR array optimization.

### 3.3. Rpl32 Is a Reliable Housekeeping Gene for Use as a Basis in ΔΔCT Analysis

**Figure 2:**
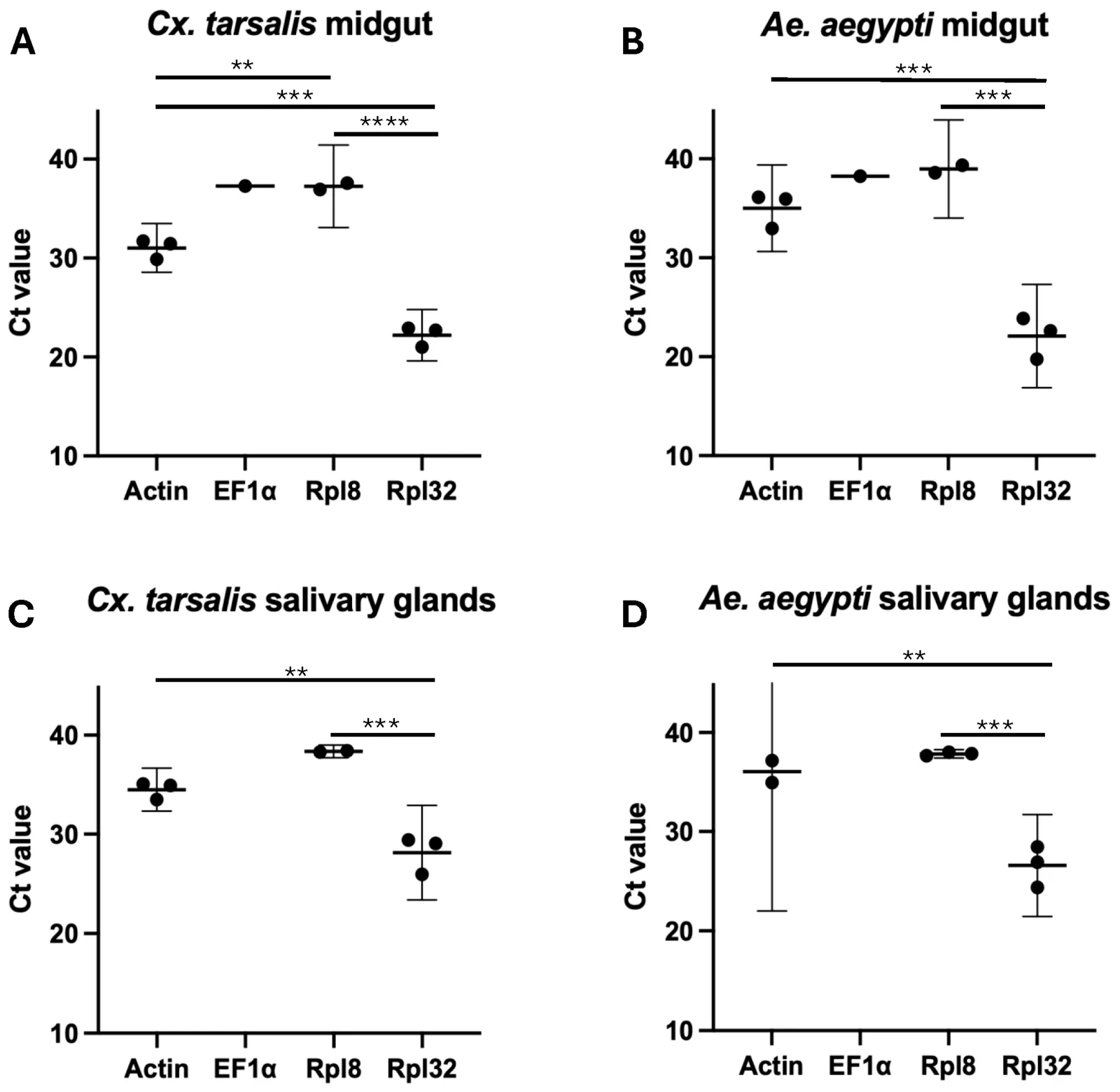
Candidate housekeeping gene detection in *Cx. tarsalis* and *Ae. aegypti* midguts and salivary glands. Bars represent 95% confidence intervals. Each species-sample type subset was analyzed by ANOVA and showed evidence of significant differences in Ct value between the candidate genes (*p* ≤ 0.001 for all groups). This analysis was followed by Tukey’s multiple comparison test to determine which candidate genes had significant differences. The results are represented by the horizontal bars and number of asterisks (*; *p* < 0.05, **; *p* <0.01, ***; *p* < 0.001, ****; *p* < 0.0001). EF1α was omitted from analyses due to lack of data, but is still shown here for informational purposes.

Of the four potential housekeeping genes that were compared, EF1α was the most unreliable candidate, with no amplification in the salivary glands and only one of three replicates positive for amplification in the midgut for each species. Because of the lack of data for EF1α, it was omitted from analyses. *Rpl8* was also unreliably detectable, with only two of three replicates positive for amplification in *Cx. tarsalis* midgut and salivary glands and *Ae. aegypti* midgut samples. In both species and tissue types, *Rpl32* amplification was reliably detected in all replicates and at significantly lower Ct values than both actin and *Rpl8* (Figure 2). Moreover, dissemination status did not change expression of *Rpl32*. While we utilized Rpl32 for analysis in this study, the choice of housekeeping gene is flexible.

### 3.4. Immune Genes in Ae. aegypti Were Downregulated at 3 and 7 Days Post-Challenge with MP12 in Both Midguts and Salivary Glands

**Figure 3:**
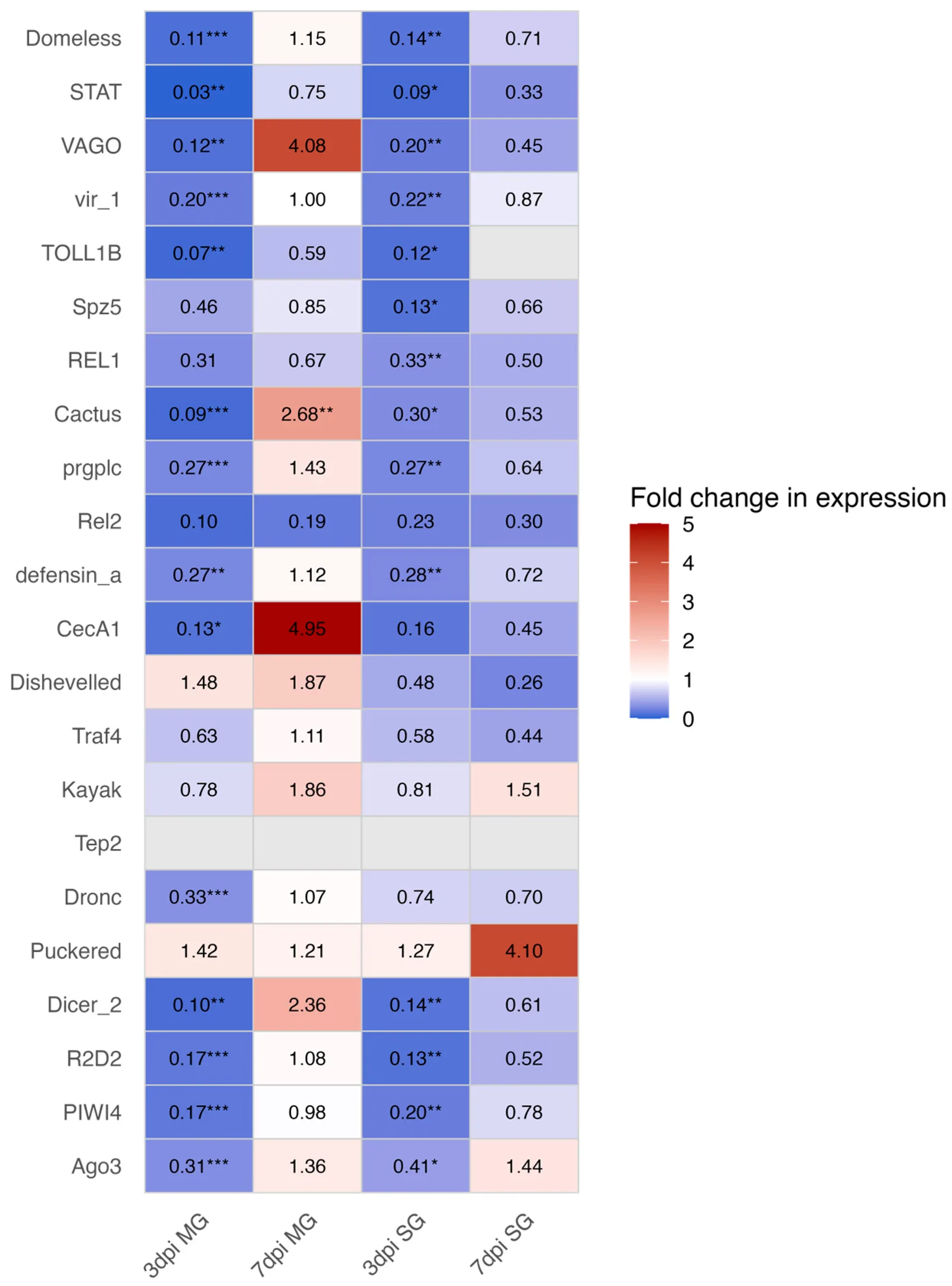
Differential immune gene expression in *Ae. aegypti* midguts and salivary glands following challenge with MP12. Mosquitoes used in this analysis were those without a detectable dissemination of RVFV MP12 compared to mosquitoes that received an uninfected blood meal. Values were determined using the ΔΔCT method of analysis with *RPL32* as the housekeeping gene. Either the Benjamini–Hochberg-adjusted *p*-value from the *t*-test or the Mann–Whitney U test was used according to the results of the Shapiro–Wilk test of normality. Significance is represented as such: *, *p* < 0.05; **, *p* < 0.01; ***, *p* < 0.001. *Tep2* was undetectable in the samples across tissues/time points. *Toll1B* was undetectable in salivary glands at 7 dpc.

At 3 dpc, *Ae. aegypti* mosquitoes with no detectable dissemination showed near-universal downregulation of the immune genes in our array (Figure 3). All genes representing the JAK-STAT and RNAi pathway were significantly downregulated, along with several genes from the IMD and Toll pathways. Interestingly, disheveled and puckered from the JNK pathway, while not significant, were the only upregulated genes at the 3 dpc time point. The midgut gene expression profile at 7 dpc exhibited several changes. The JAK-STAT antiviral response gene vago and IMD antiviral response gene cecropin A1 were both more than fourfold upregulated, though this change was not significant. Cactus, the negative regulator of the Toll pathway, was significantly upregulated at this time point. Additionally, components of the JNK and RNAi pathways were moderately, though not significantly upregulated.

At 7 dpc, most immune genes remained downregulated in the salivary glands with a few exceptions, though none of the changes in expression at this time point was statistically significant. Puckered, a negative regulator, remained slightly upregulated in the midgut. Interestingly, the JNK pathway transcription factor kayak was slightly upregulated (1.51-fold) and the JNK pathway inhibitor puckered upregulated (4.1-fold) at 7 dpc in the salivary glands. *Ago3*, an RNAi response enzyme that generates piRNAs, was also slightly upregulated (1.44-fold) in the salivary glands at 7 dpc.

### 3.5. Culex tarsalis Responded to MP12 Challenge with Early Immune Gene Upregulation That Reduced by 7 dpc

**Figure 4:**
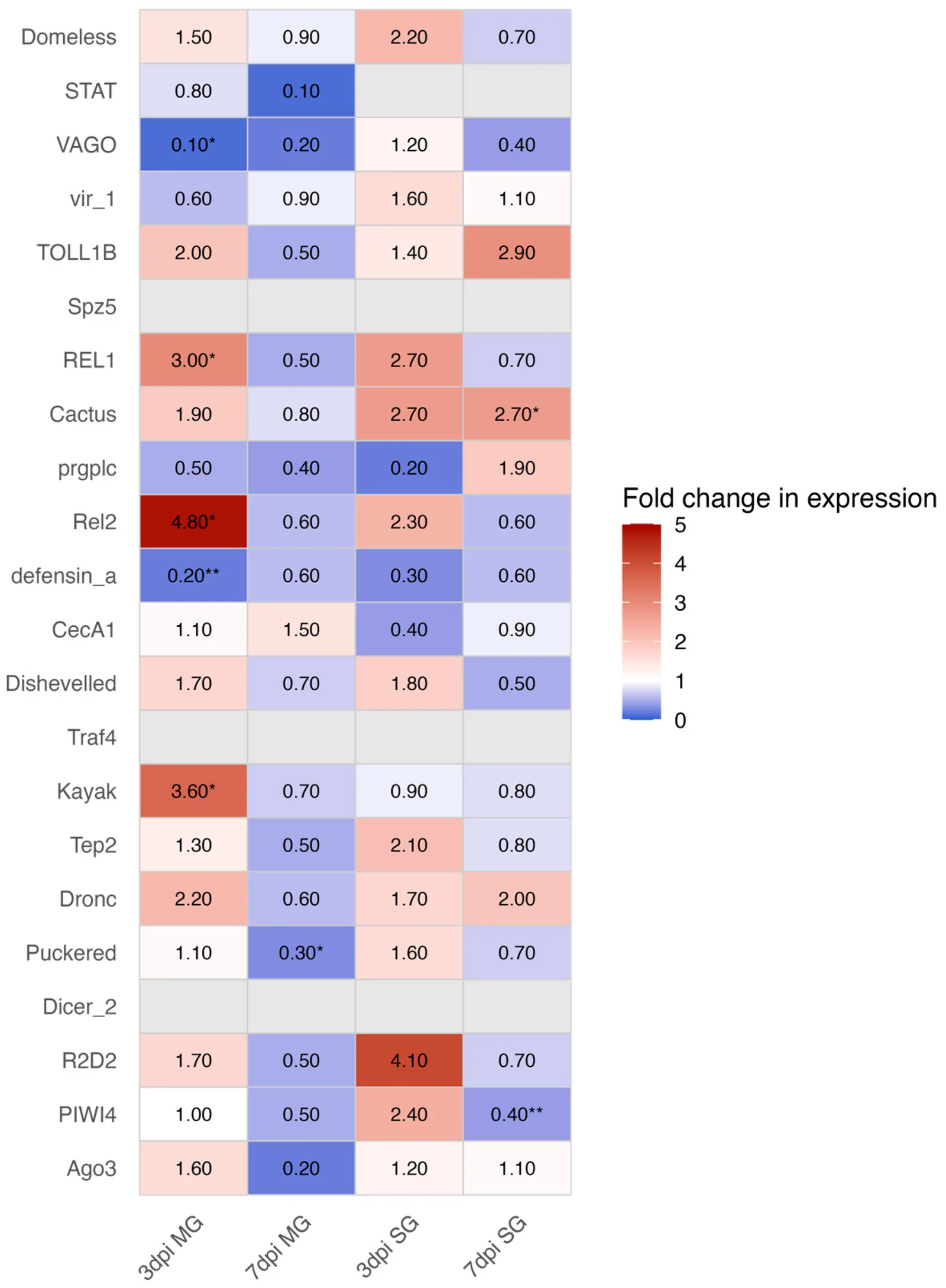
Differential immune gene expression in *Cx. tarsalis* midguts and salivary glands following challenge with MP12. Mosquitoes used in this analysis were those with detectable dissemination of RVFV MP12 compared with those that received an uninfected blood meal. Values were determined using the ΔΔCT method of analysis with *Rpl32* as the housekeeping gene. Either the Benjamini–Hochberg-adjusted *p*-value from the *t*-test or the Mann–Whitney U test was used according to the results of the Shapiro–Wilk test of normality. Significance is represented as such: *, *p* < 0.05; **, *p* < 0.01; ***, *p* < 0.001. *Dicer 2* and *Traf4* were undetectable/unreliable in control samples, and thus ΔΔCT could not be calculated. *Spaetzle 5* was undetectable in all samples. STAT had unreliable detected in salivary glands.

In both the midgut and salivary glands of *Cx. tarsalis* at 3 dpc, there was gene upregulation across all examined immune pathways in virus-challenged mosquitoes compared to unchallenged mosquitoes (Figure 4). The only JAK-STAT pathway component that was upregulated was domeless, the cytokine receptor-like transmembrane receptor that activates the pathway upon binding of the cytokine-like protein unpaired. The other pathway components, including the antiviral peptides vago and *Vir1* were downregulated, though not significantly. Toll pathway components were upregulated, with the NFκB transcription factor *Rel1* expression significantly upregulated threefold in the midgut. Of the IMD pathway genes, the receptor *PRGP-lc* appeared to be downregulated and the response genes defensin A and cecropin A1 were significantly downregulated and not differentially expressed in challenged mosquitoes, respectively. Interestingly, the IMD pathway transcription factor *Rel2* was significantly upregulated 4.8-fold in midguts of challenged mosquitoes. The JNK pathway components disheveled, Kayak, and Dronc were upregulated in the midgut, though not significantly. *Traf4* was consistently undetectable in uninfected control mosquitoes, but detectable in MP12-challenged mosquitoes. While the lack of a control baseline precludes formal analysis, this could indicate that *Traf4* is being upregulated to detectable levels in MP12-challenged mosquitoes. Similarly, in the RNAi pathway, Dicer 2 was not reliably detected in uninfected control mosquitoes (precluding ΔΔCT calculating) or in some MP12-challenged mosquitoes, but interestingly all 3 dpc midgut samples had Dicer 2 detection with an average CT of 33. *R2D2* and *Ago3* were moderately upregulated RNAi components, while *PIWI4* expression remained unchanged. The salivary glands showed a similar pattern in the examined pathways, though the values were not statistically significant. A notable difference is an apparently more dramatic upregulation of RNAi pathway components *R2D2* and *PIWI4*.

At 7 dpc, the genes that were upregulated in the midgut at 3 dpc were downregulated compared to uninfected controls, though only the downregulation of STAT, vago, and puckered were statistically significant. The trend in the salivary glands was similar, though *Toll1B* was upregulated and cactus, the inhibitor of the Toll pathway, was significantly upregulated. The Toll pathway has previously been shown to control dengue virus infection in *Ae. aegypti* mosquitoes [23,24]. Additionally, the IMD pathway receptor PRGP-lc was upregulated and the apoptotic signaling protein Dronc was upregulated, though not significantly.

Overall, most genes assessed were significantly downregulated in *Ae. aegypti* midguts with no detectable dissemination and salivary glands in response to MP12 challenge compared to *Cx. tarsalis* with detectable disseminations (Figure 3 and Figure 4).

### 3.6. Immune Gene Expression Was Distinct Between Ae. aegypti and Cx. tarsalis Following Oral Challenge with MP12

**Figure 5:**
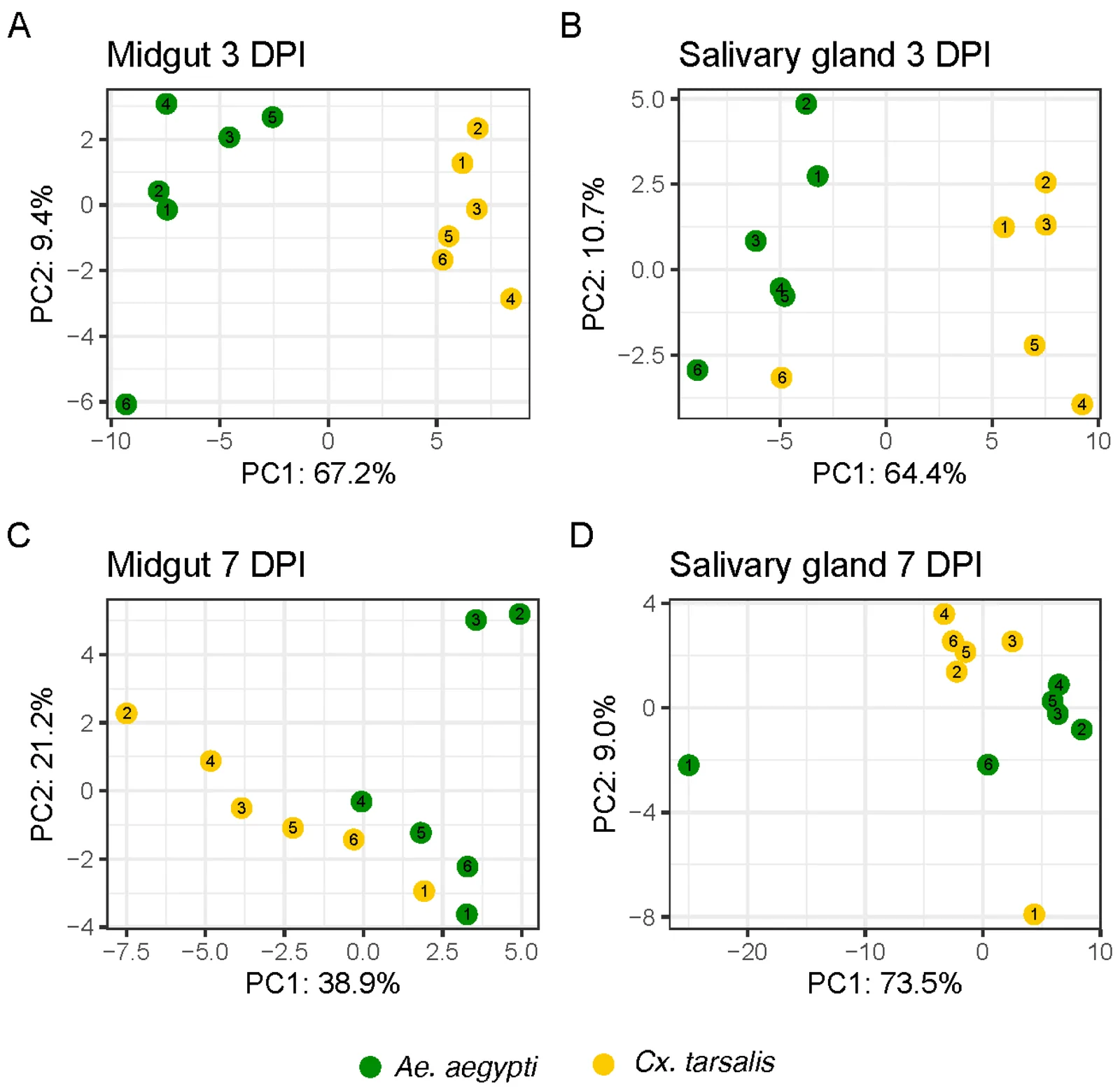
Principal component analysis of normalized qPCR expression values for midgut and salivary gland tissues at 3 and 7 days post-challenge. Each point (colored dot with a number) represents an individual sample, and PC score is based on a linear combination of the ΔΔCt values for that sample.

Principal component analysis (PCA) of normalized qPCR expression values revealed separation of samples by species across both tissues and time points (Figure 5). In all conditions, samples clustered primarily by species along PC1, which explained 38.9–73.5% of the total variance, with the weakest separation in midguts at 7 dpc (Figure 5C). This pattern indicates that species identity is a major contributor to overall variation in gene expression profiles in both midgut and salivary gland tissues following an oral challenge with MP12.

**Figure 6:**
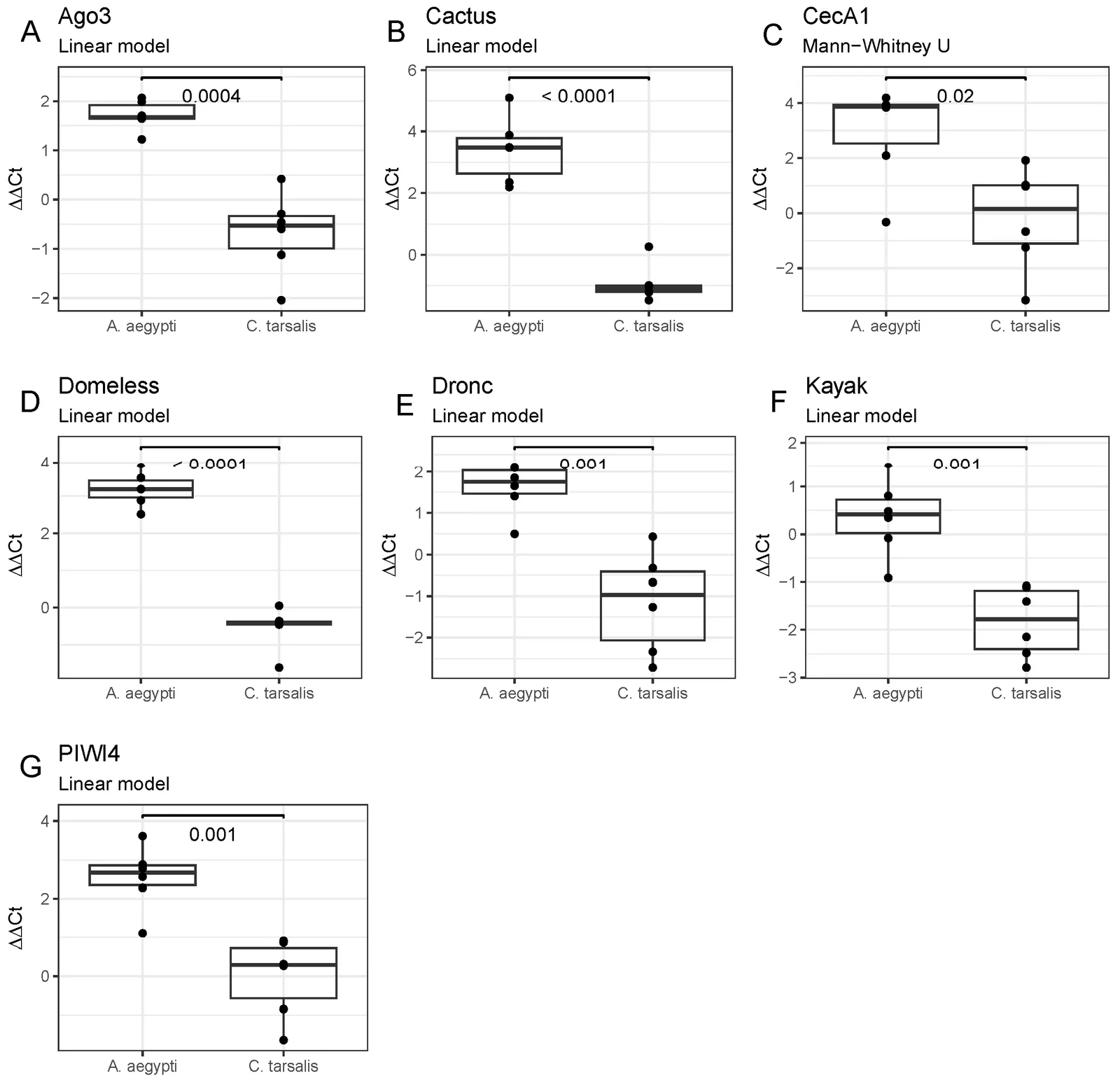
Differential gene expression of immune genes from interspecies comparison of *Ae. aegypti* and *Cx. tarsalis* midguts at 3 dpc. *p*-values are either from linear models or Mann–Whitney U tests, as shown in the plot subtitle.

**Figure 7:**
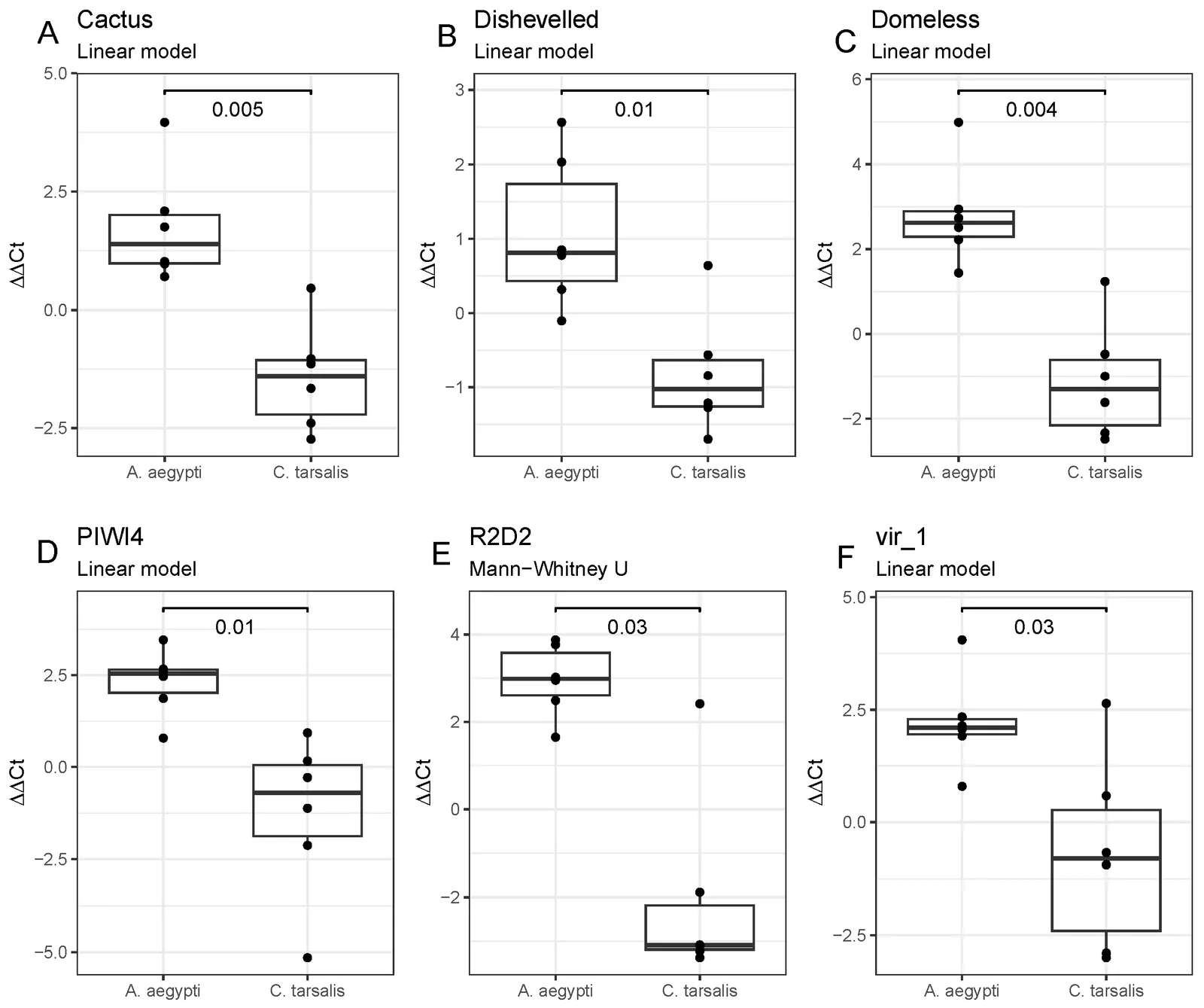
Differential gene expression of immune genes from interspecies comparison of *Ae. aegypti* and *Cx. tarsalis* salivary glands at 3 dpc. *p*-values are either from linear models or Mann–Whitney U tests, as shown in the plot subtitle.

**Figure 8:**
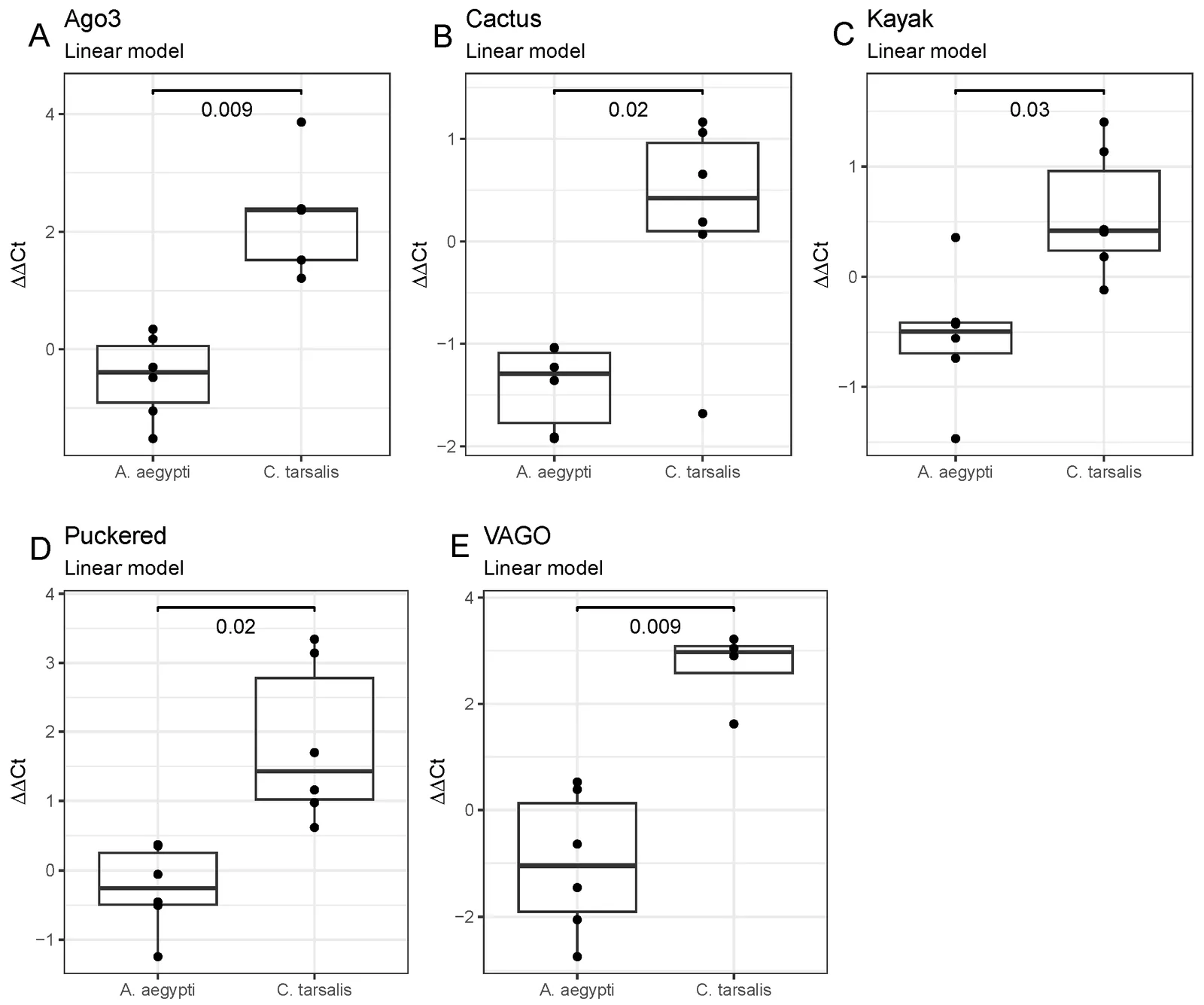
Differential gene expression of immune genes from interspecies comparison of *Ae. aegypti* and *Cx. tarsalis* midguts at 7 dpc. *p*-values are either from linear models or Mann–Whitney U tests, as shown in the plot subtitle.

Examination of individual gene expression changes at 3 dpc revealed a pattern of immune gene downregulation in *Ae. aegypti* when compared to *Cx. tarsalis*. In the midgut at 3 dpc, there were genes across the five immune pathways that were significantly more downregulated in *Ae. aegypti* than in *Cx. tarsalis* (Figure 6). In the salivary glands at 3 dpc, the IMD pathway was not differentially regulated when comparing the two species (Figure 7), though again the other pathways had components that were significantly less expressed in *Ae. aegypti*. The gene expression profiles of the midguts at 7 dpc shows a reversal of this pattern, with genes in the JAK/STAT, Toll, Jnk, and RNAi pathways being significantly upregulated in virus-challenged *Ae. aegypti* compared to *Cx. tarsalis* (Figure 8). There were no significant interspecies differences in regulation of the examined genes in the salivary glands at 7 dpc. Since the dissemination phenotype was different between these mosquito species, further investigation of the potential involvement of these immune genes in modulating vector competence is warranted.

## 4. Discussion

The development of a qPCR array for use in multiple key mosquito vector species represents an important contribution to the toolkit for studying virus–vector interactions in a variety of arbovirus systems. The industry standard for examining differential gene expression is RNAseq; however, this method has limitations that reduce its accessibility and applicability for some projects. RNAseq can take several months from the point of experiment to analysis and requires significant computational resources that may not be available to all research groups. Additionally, mosquito tissue samples typically must be pooled to provide enough input for RNAseq, eliminating the ability to analyze gene expression on an individual basis.

To circumvent these challenges, we developed a new qPCR array to examine the immune response of two mosquito species orally challenged with RVFV MP12 at the two major physical barriers: the midgut and the salivary glands. This qPCR array can be applied to single tissues from individual mosquitoes, and the data can be acquired and analyzed within days of sample collection without significant computational skills or resources. Moreover, primers were developed for *Cx. tarsalis*, *Cx. quinquefasciatus*, and *Ae. aegypti*, allowing for comparative analyses and application to all three vector species.

As a pilot test of this array, we challenged both *Ae. aegypti* and *Cx. tarsalis* mosquitoes with RVFV MP12 and analyzed midguts and salivary glands at 3- and 7 dpc. Species identity was a major contributor to the variation observed in immune responses (Figure 5), but some observations may explain the differences in dissemination rate between the species (Table 1). While at 3 dpc, the dissemination rate was not significantly different, the dissemination rate on day 7 post-challenge was significantly lower in *Ae. aegypti* compared to *Cx. tarsalis* (26.7% and 65%, respectively; *p* = 0.041), indicating that this is an informative time point for studying differences in the midgut barriers relevant to vector competence.

In this evaluation, *Rpl32* was the most reliable housekeeping gene of the four genes evaluated. *Rpl32* has been widely used as a housekeeping gene for insects, including mosquitoes [25,26,27,28,29]. Use of this housekeeping gene has not always been stable and may vary by life stage [25]. Investigators may wish to evaluate and use a different housekeeping gene, since this platform is flexible towards that choice.

*Culex tarsalis* is a highly susceptible vector with a weak midgut infection, moderate midgut escape, and moderate-to-weak salivary gland barrier [14,15,21]. In contrast, *Ae. aegypti* has a high midgut infection barrier and moderate-to-weak midgut escape and salivary gland barriers [21,30]. These trends are consistent with the observations in this study in which the virus dissemination rate was 65% by 7 dpc in *Cx. tarsalis* but only 26.7% in *Ae. aegypti* (Table 1). The qPCR array revealed a pattern of immune gene up- and downregulation across the different pathways in *Cx. tarsalis* midguts and salivary glands at 3 dpc with RVFV-MP12 that provides potentially interesting insights considering the vector competence profile of this species and provides a basis for hypothesis development. Some of the genes with significant differential expression soon after virus challenge include downregulation of antiviral effectors, including vago, vir-1, defensin, and cecropin A. The suppression of these genes by 3 dpc may contribute to the successful establishment of a midgut infection in this highly competent vector while working in concert with regulatory elements in other pathways that keep virus infection in check. While most immune genes in the JAK/STAT and IMD pathways were downregulated, the Toll pathway and the JNK pathway were upregulated, suggesting an early antiviral role for these pathways in the midgut. Notably, *Rel2* was significantly upregulated. *Rel2* has been demonstrated to be upregulated in response to the presence of reactive oxygen species, and is protective during CHIKV infection of *Aag2* cells [31]. Therefore, we speculate that *Rel2* could be functioning to protect the integrity of midgut epithelial cells where virus replication is actively occurring. The upregulation of *Rel2* and kayak was dampened by 7 dpc in both tissues and especially in the midgut, indicating that their primary role seems to be early in infection. Interestingly, *PIWI4* and *R2D2* were both upregulated in the salivary glands at 3 dpc. Tassetto et al. [32] found that PIWI elements regulated expression of endogenous viral elements and primed the immune response in such a way to help control active infection. This response so early on provides evidence for systemic responses of mosquito tissues to the presence of a virus. The role of immune gene expression in the salivary glands so early after exposure warrants further investigation. Collectively, the balance of up- and downregulation of genes in different pathways appears to be a response to cellular damage caused by viral infection. Effector molecules that may have been protective were suppressed early on, and the observed upregulated genes may be functioning to protect cells from damage during virus infection. Some pathways that could function to limit virus replication were not significantly upregulated. The short-lived upregulation of these pathways may indicate immune modulation by the virus. While interesting to review and develop hypotheses from, each of these preliminary results will need to be tested with functional validation work to characterize the role of these factors in modulating virus infection in mosquitoes.

In contrast to *Cx. tarsalis* with detectable disseminations, *Ae. aegypti* without detectable disseminations responded to challenge with near-global downregulation of the analyzed genes at 3 dpc followed by selective upregulation in the midgut, but continued downregulation in the salivary glands at 7 dpc. We hypothesize that the immune downregulation at 3 dpc may indicate a strategy of immune tolerance in *Ae. aegypti*. The minimizing of the immune response may preserve the integrity of the physical barrier in the midgut by preventing cellular damage. Conversely, it is possible that *Ae. aegypti* mosquitoes have higher baseline expression of innate immune genes that effectively prevent the establishment of infection. Sim et al. [33] used transcriptomics to quantify basal-level immune activation among diverse populations of *Ae. aegypti* and found that it was negatively correlated with dengue virus susceptibility. Moreover, the strong vs. weak midgut infection barrier in *Ae. aegypti* compared to *Cx. tarsalis* may be reflected in the later timing of when significant expression of particular genes was observed. Since the qPCR array used in this study relies on relative quantification, a method of absolute quantification would be necessary to determine if baseline expression differs between *Ae. aegypti* and *Cx. tarsalis*.

By 7 dpc, the strong downregulation in the midgut appears to have normalized and the upregulation of antiviral effectors vago and cecropin A1 suggests a switch to a response strategy rather than a tolerance strategy. Kayak was upregulated in both tissues at 7 dpc. Previous work has found that RNAi silencing of the JNK pathway transcription factor Kayak increases the infection rate and intensity of flaviviruses in *Ae. aegypti* mosquitoes, indicating a protective role for this pathway during viral challenge [34,35,36].

## 5. Conclusions

Here, we report the development of a novel qPCR gene array that is broadly applicable to studies focused on mosquito immune responses to pathogens. Primers were designed for three key mosquito vectors, making this a powerful new tool to compare mosquito immune responses within and between vector species, in different tissues, and for individual as well as pooled tissues. This qPCR array was applied during a pilot test of *Ae. aegypti* and *Cx. tarsalis* infected with the RVFV MP12 strain to assess the utility of the assay and provide some preliminary insights into immune gene expression in these mosquito species infected with this virus strain. Gene expression patterns during two scenarios were evaluated: *Ae. aegypti* with no detectable disseminations, and *Cx. tarsalis* with detectable disseminations of RVFV MP12. These findings do not necessarily mean that immune responses against wild-type strains of RVFV would be the same. Assessment of mosquito immune responses against wild-type RVFV should be a separate, future study. Some additional limitations to this approach are that only selected genes from each pathway are being analyzed, which provides an incomplete view into the immune responses. While these results provide a valuable snapshot across all immune pathways at the prescribed time points and tissues, these results may be missing some critical components. In this case, results are still interpretable to the level of the pathway and could be used to generate more specific hypotheses to follow up with more comprehensive approaches.

## Figures and Tables

**Table 1:** Number of mosquitoes positive for MP12 dissemination by RT-qPCR of legs and wings; *p*-values represent results of Fisher’s exact test comparing the rate of dissemination between the two species at each time point.

Days Post-Challenge	*Ae. aegypti* (n Positive/n Total)	*Cx. tarsalis* (n Positive/n Total)	*p*-Value (Fisher’s Exact Test)
3	2/15 (13.3%)	4/20 (20%)	0.680
7	4/15 (26.7%)	13/20 (65%)	0.041

## Data Availability

The original contributions presented in this study are included in the article/supplementary material. Further inquiries can be directed to the corresponding author(s). The raw data supporting the conclusions of this article and additional primers for *Cx. quinquefasciatus* are available through Zenodo: DOI 10.5281/zenodo.20058272.

## Supplementary Materials

The following supporting information can be downloaded at https://www.mdpi.com/article/10.3390/v18080818/s1. Table S1: Housekeeping gene primer sequences; Table S2: qPCR array primers; Table S3: Gene IDs used for alignment and primer design.
