# Supplementary material for: Development of a Novel Immune Gene Array to Assess Mosquito Gene Expression During Arbovirus Infection
Source: Viruses. 2026 Jul 25;18(8):818. doi: 10.3390/v18080818 (PMC13517103; doi:10.3390/v18080818)
Supplement: Supplementary file 1 [file viruses-18-00818-s001.zip › viruses-4396424-supplementary.pdf]

Supplementary Materials

Supplementary Table S1. Housekeeping gene primer sequences

Gene	Organism	Forward primer	Reverse primer
Actin	*Ae. aegypti*	GTCTCCGGGCTGTTCTATATTC	GGCAACAATCCGAGCTAAAC
Actin	*Cx. tarsalis*	GCTTCGTGTCGCAGGATTTC	ATGATGGTGTTGTCCGGGTG
EF1α	*Ae. aegypti, Cx. tarsalis*	CCTTGAACCAGGGCATCTT	AGGAAGTCTCGTCCTACATCA
RPL8	*Ae. aegypti, Cx. tarsalis*	AAGTACAAGGTGAAGCGTAACT	GCCTTACCGATGTGCTGAT
Rpl32	*Ae. aegypti*	GCGATTTCGGCACAGTAGA	GGCTTCAAGAAGTTCCTGGT
Rpl32	*Cx. tarsalis*	ACCAGGAACTTCTTGAAGCC	CAGTACCTGATGCCGAACAT

Supplementary Table S2. qPCR array primers

Gene	*Ae. aegypti* FWD' Sequence	*Ae. aegypti* REV' Sequence	*Cx. tarsalis* FWD' Sequence	*Cx. tarsalis* REV' Sequence
Domeless	GGA TAT GTC CAG GCA CCT TTA G	TTT CGA GAG GCT CGG ATT ATT G	GCGACCGGATTCCAGTATAAG	CTGGATCATCGTGCTTCCAT
STAT	ACC TTC TGG GAG TGG TTC TA	CGG CTT TAG ACT TGT GGA TGA	ACC TTC TGG GAG TGG TTC TA	CGG CTT TAG ACT TGT GGA TGA
VAGO	CTC GAT CCC GAT GAA GAG AAA TC	CGA CAG TAC ACA GCT CGT AAA G	CGTGAACGATCCTCACTGC	ACTTGTACTTGTGGCAACACT
Vir-1	CAG TGG AAA CTG GAA ATC GTA ATG	GCC TCA GTA GTT GGT TCC TTA TT	CAAGAAGGACTCCGATGATGAG	CTCGAGTCCAGCTCGGT
TOLL1B	CGA GCC TAA AGT CAG GGA ATA C	TTT CCA CTC CCA GGA AAC TAA C	GATCCGTACACTTCGCTCAC	GACAAGTCCAGGAAGCTCAAA
Spaetzle 5	CAAGCGAGTGCTCCAATCT	ACGTACAAACTCTGCCCATC	AAACTGGATGTACGTGGTCAA	TTC GGC AGG ATG CAC AG
REL1	CCG GTG GCA GAG AGA TTA TTT	TTC TTC CCA CAC GAT GTT ACC	GAG CTA CAC GTT CAG CAA TCT	ATC CAC CCG GAT CTC CTC
Cactus	TGA AGT CCA AGG AGC AAC AA	CGG CAA GGT GTA GGT AAG TAT C	CCG ATG TCG TTC TGG ATG TC	TAC CTC CAT CTG GCC ATC A
PGRP-LC	CAT CGT CCA CTA GTG CTC TAA C	AGT TGT GTC GGC CTT ATC TAT C	GACACATCGCCACCAACTA	GGTTCTCGAAGAACGCATTTC
Rel2	CGG TTA CTG GAG GAC TAT TCA C	CAG ATA TAC GGA GCC ATC TTC C	CGGTGCCCTTCAAGTACAA	TCGGAATCTCGCTGGAAATG
Defensin A	GAT TCG GCG TTG GTG ATA GT	GAT TCG GCG TTG GTG ATA GT	TCG ACG AAC TCC CAG AGG	AAA TCC ACT TAG CAG GTC ACA G
CecA1	TGC ACT CGT TCT GCT CAT C	TCC GAC TCC TTC CAA TTT CTT C	CTGTTCGTAATCGTCCTGCT	GCTTCTTGCCGAACTTCTTG
Dishevelled	CCA GCT CCG AAC CAT GAA TAA	TCC AAA TCG CTG GAC ACT AC	TGCCAAATCCACCGATCTAC	CAGTTCCGACATCCTAACCAG
Traf4	CTT CCT CTC ATC TGT CGC TAA TG	CGG TGT AGT TGG TAC TGA GTT T	CACGTGTCCGTCTACATCAA	CTGCTCGAAGAGGGTGAAG
Kayak	AGT ATC GCG CCT AAA CCA TC	CCT ACC GCA CTG TTG CTA TT	TCGCCTCCTTCGATGGTAT	GAACGTCTGCTCGATGTTCTT
TEP2	GAGCAGAACATGCTGAACTTTG	CTGATAGCCGGACTCCATAAAC	GAGCAGAACATGCTGAACTTTG	CTGATAGCCGGACTCCATAAAC
Dronc	CCAGTTACCTATCGACGGAATG	CGACGTATAGGACGGAATTATCG	AATGATCAACCGGAACAAGGA	ACCTGGCACATGCTTTCA
Puckered	GCACAAACGGCACAAAGATAC	GATGCGATTCGATGTCGTAGG	GCACAAACGGCACAAAGATAC	GATGCGATTCGATGTCGTAGG
Dicer 2	AACGCACGCTATCCAGTATC	GCAATTCGCAGAACGTAGATG	GGCAGGTGGTGAAGGATTT	GCGATACCAGGGAAGAATGAC
R2D2	GACGAATTGCTCTGGACGAA	CGCTGTTGTCTCGTTTCATTTC	GAGCACATCATTCGCAACTATC	TCCTGGCAGCGTCAATTT
PIWI4	TCTTCGGAGGATTCCTGTTTG	AGTACGTTCGTGTCTGGATTG	AGCTGGTCGGACGTAATCT	CGTGCTGACGAATACTGGTAATG
Ago3	CCAAGACAAACCGATGGAAATG	CGATGTCTCTCATCACCTTGAA	CGCATCGACGACATCATCTT	GTACTGCTGCTTGTAGTACTCG
Rpl32	GCGATTTCGGCACAGTAGA	GGCTTCAAGAAGTTCCTGGT	ACC AGG AAC TTC TTG AAG CC	CAG TAC CTG ATG CCG AAC AT

Supplementary Table S3. Gene IDs used for alignment and primer design.

Pathway	Function	Gene	Cx. quinquefasciatus gene ID (VectorBase)	Cx. tarsalis gene ID (Main *et al* 2021)	Ae. aegypti gene ID (NCBI)
	Receptor	Domeless	CPIJ017416	gene1501	AAEL012471
	Intermediate	STAT	CPIJ016470	gene11364	AAEL020559
	Response Gene	VAGO	CPIJ000699	gene2317	AAEL000200
JAK-STAT	Response Gene	Vir-1	CPIJ012373	gene2062	AAEL018117
	Receptor	TOLL1B	CPIJ006515	gene6385	AAEL013441
	Ligand	Spaetzle 5	CPIJ009906	gene8864	AAEL001929
	Intermediate	REL1	CPIJ002469	gene5677	AAEL007696
	Response Gene	Defensin A	CPIJ001276	gene5564	AAEL003841
Toll	Inhibitor	Cactus	CPIJ004774	gene6092	AAEL000709
	Receptor	PGRP-LC	CPIJ000672	gene4218	AAEL014640
	Intermediate	Rel2	CPIJ012236	gene3946	AAEL007624
IMD	Response Gene	CecA1	CPIJ010699	gene10089	AAEL025975
	Receptor	Dishevelled	CPIJ016589	gene9899	AAEL008234
	Ligand	Traf4	CPIJ005312	gene12410	AAEL006649
	Intermediate	Kayak	CPIJ003767	gene12112	AAEL008953
	Response Gene	Dronc	CPIJ002923	gene13123	AAEL011562
JNK	Inhibitor	Puckered	CPIJ008018		AAEL006134
	siRNA: Rnase	Dicer 2	CPIJ010534	gene9527	AAEL006794
	siRNAL dsRNA binding	R2D2	CPIJ011746	gene6258	AAEL011753
	piRNA: Rnase	PIWI4	CPIJ002459	gene2470	AAEL007698
RNAi	piRNA: dsRNA binding	Ago3	CPIJ005275	gene3988	AAEL007823
Housekeeping	Basis for ΔΔCT analysis	Rpl32	CPIJ001220	gene5028	AAEL003396
		Rpl8	CPIJ000162	gene5805	AAEL000987
		EIF1α	CPIJ009303	gene1055	AAEL017096
		Actin	CPIJ016462	gene2985	AAEL001673
